# The Accumulated Effect of the Number of Ethylene Oxide Units and/or Carbon Chain Length in Surfactants Structure on the Nano-Micellar Extraction of Flavonoids

**DOI:** 10.3390/jfb11030057

**Published:** 2020-08-07

**Authors:** Karolina Śliwa, Paweł Śliwa

**Affiliations:** Faculty of Chemical Engineering and Technology, Cracow University of Technology, 24 Warszawska St., 31-155 Cracow, Poland; karolina.sliwa@pk.edu.pl

**Keywords:** ethoxylated fatty alcohols, ultrasonic assisted micelle-mediated extraction, dynamic light scattering, molecular dynamic simulation, flavonoids

## Abstract

Classical extraction methods used for isolation of active substances from plant material are expensive, complicated and often environmentally unfriendly. The ultrasonic assistance micelle-mediated extraction method (UAMME), based on green chemistry principles, seems to be an interesting alternative. This work aimed to find a connection between the chemical structure of non-ionic surfactants and the efficiency of the extraction process. The effect of hydrophobic chain length and number of ethoxy groups on the quality of *Bidens tripartite* extracts was investigated. Several ethoxylated fatty alcohols were used: Ceteareth-20, Steareth-20, Oleth-20, Oleth-10, Oleth-5, C12-C13 Pareth-12, C12-C15 Pareth-12 and Ceteareth-12. The bioflavonoid compositions with the HPLC method was determined. The hydrophilic lipophilic balance (HLB) of studied surfactants, as well as the surface tension of surfactant solutions, were compared, to determine the explanation for the obtained differences in bioflavonoids concentration. The structural changes influenced by polyphenol extraction were monitored using Dynamic Light Scattering (DLS) measurements. In this work, probably for the first time, the connection between the chemical structure of non-ionic surfactants and the efficiency of the extraction process was found. The experimental and theoretical approach rationalized the choice of an appropriate eluent. We propose some structurally dependent factors, whose optimal value gave a high efficiency to the UAMME.

## 1. Introduction

Solubilization is the spontaneous formation of a stable, isotropic solution of an insoluble or slightly water-soluble substance in an aqueous micellar solution. The solubilization process occurs because an aqueous solution of surfactants forms macroscopically homogeneous, but microscopically heterogeneous, microemulsions [1,2]. The micellar solubilization is very useful, for example, in pharmacy, because many active substances do not dissolve or dissolve very poorly in water. Furthermore, this method protects active substances against degradation, increases their bioavailability and minimizes their side effects. The solubility of the active substance is low when the surfactant concentration is below its critical micellar concentration (CMC). Above CMC, solubility increases linearly with increasing surfactant concentration [3]. The solubilization process, often called micellar-mediated extraction (MME), occurs mainly through hydrophobic interactions between the extracted substance and surfactant molecules. In addition, electrostatic interactions, especially the formation of hydrogen bonds are important [4,5,6].

The location of the solubilizate in the micelle depends mainly on its nature [1]. For both non-ionic (e.g., the ethoxylated/propoxylated alcohols) and ionic surfactants, the hydrophilic substances are solubilized on the micelle surface. Compounds with moderate hydrophobicity place them into the polyether shell or between the hydrophilic heads and the first few carbon atoms of the hydrophobic chains (i.e., outer core). Substances completely insoluble in water are put inside the lipophilic core of micelles [3,7,8].

The efficiency of the solubilization depends on the surfactant structure, solubilizate structure, process temperature and pH of the solution. Generally, non-ionic surfactants seem to be better solubilizing agents, due to the lower critical micellar concentrations, less toxicity and major effectiveness at low concentrations [1]. In some cases, the use of a mixture of non-ionic and ionic surfactants seems to be beneficial. That approach causes an increase in the extraction efficiency of polar organic compounds [9]. The solubilization yield of weakly polar molecules, such as long-chain hydrocarbons, usually increase with lengthening hydrophobic surfactant chains [1,10]. However, the longer the hydrophobic chain of the solubilizate molecules, the less efficient extraction. It has been proven that unsaturated compounds are more easily extracted than their saturated analogues [1]. The solubilization of aromatic compounds is usually greater than aliphatic ones [3]. The structure of the hydrophilic part of surfactants also affects the solubilization process. In the case of alcohol ethoxylates, the phenol solubilization equilibrium constant increased linearly with EO number and was unaffected by alkyl carbon number or hydrophobe branching [11].

In our previous studies [6,12], the ultrasonic-assisted micelle-mediated extraction methodology (UAMME) was used to obtain the extracts of *Bidens tripartite* L. In an earlier study [12], aqueous solutions of different types of non-ionic surfactants (ethoxylated/propoxylated stearyl alcohol, octylphenol ethoxylate, cetearyl glucoside, sucrose stearate) and whey protein concentrates were utilized as the extraction solvents. UAMME extracts were rich in chlorogenic acid, caftaric acid and its derivatives together with luteolin 7-O-glucoside. The concentration of particular active substances varied depending on the kind of surfactant. The luteolin glucoside was extracted only using ethoxylated alcohols. In a second work [6], polyethylene/polypropylene glycol ethers of fatty alcohols were utilized for solubilization and concentration of *Bidens tripartite* flavonoids. The main constituents of the extracts were chlorogenic acid and di-O-caffeoylquinic acid. The highest concentrations of each of the flavonoids, as well as their total concentration, were obtained in the case of the most hydrophilic surfactant (Rokanol^®^ NL5, PCC Group, Brzeg Dolny, Poland). Furthermore, the results revealed that the dicaffeoylquinic acid, the most hydrophobic compound, was easiest solubilized into the surfactant pseudo-phase, compared to other polyphenols. The increase in the aggregate size during the micelle-mediated extraction was observed by DLS measurements. The results of molecular dynamics simulations indicated that the amphiphilic properties of the polyphenols force compounds to place them between the hydrophilic shell and the hydrophobic core of the surfactant micelle. The chemical structure of the particular compound rather than its logP value should control the configuration of the micelle-polyphenol complexes.

However, there are some gaps in the above work that disturb the quantitative assessment of the dependence between flavonoid solubilization efficiency and the surfactant structure modification. Therefore, several surfactants with a systematically variable structure element were used in this work. Effectiveness of eight non-ionic surfactants: Ceteareth-20, Steareth-20, Oleth-20, Oleth-10, Oleth-5, C12-C13 Pareth-12, C12-C15 Pareth-12 and Ceteareth-12 in the extraction of *Bidens tripartite* herb was evaluated. The dynamic light scattering (DLS) measurements were used to observe the structural changes accompanying the solubilization of flavonoids by surfactant solutions. Additionally, the structure of the luteolin glucoside incorporation into the surfactant micelle was theoretically simulated using a molecular dynamic method. The use of both theoretical and experimental methodology is very desirable and often provides additional information and helps to interpret the results [5,6,13,14].

## 2. Materials and Methods

### 2.1. Materials and Chemicals

Sodium hydroxide, sodium nitrite and aluminium chloride were purchased from POCh (Gliwice, Poland). The Folin-Ciocalteau’s reagent, as well as HPLC-grade standards chlorogenic acid and luteolin-7-O-glucoside, were bought from Sigma Aldrich (Poznań, Poland). The used surfactants, i.e., Ceteareth-20 (BrijTMCS20), Steareth-20 (BrijTMS20), Oleth-20 (BrijTMO20), Oleth-10 (BrijTMO10), Oleth-5 (BrijTMO05), C12-C13 Pareth-12 (Brij TMLT12), and Ceteareth-12 (Brij TMCS12), were kindly supplied from Croda Personal Care (Croda Poland sp. zo.o., Kraków, Poland) and C12-C15 Pareth-12 (Tomadol 25-12) from Air Products (Allentown, PA, USA). *Bidens tripartite* (three-part beggarticks) herb, from Dary Natury (Koryciny, Poland) was used in the extraction process. The same plant material was used in a previous work [6]. If not specified, reagents were of analytical grades.

### 2.2. Characteristics of Surfactants

The surface tension of 1% surfactant solutions and their critical micelle concentration (CMC) was measured at 25 °C, using the STA1 tensiometer equipped with the thermostat Huber ministat125 and the Sartorius analytical balance. Cloud points of 1% water solutions of surfactants were measured using Macherey Nagel Nanocolor spectrophotometer. Measurements were made in triplicate and turbidity was recorded as the ratio of Nephelometric Turbidity Units/Formazin Nephelometric Units (NTU/FTU). The Hydrophilic/Lipophilic Balance factor (HLB) was calculated using InstantJChem software [15].

### 2.3. The Extraction Procedure

Dried *Bidens tripartite* herb was deeply extracted with 200 mL of distilled water, 96% *v*/*v* ethanol or 1% *w*/*v* aqueous solutions of the surfactants. In all cases, 3 g of the plant’s raw material was exactly weighed. To ensure proper mixing, the extraction process was performed using an ultrasonic bath (50 Hz, 300 W), for 30 min, at 25 °C.

### 2.4. Determination of Polyphenol Content in the Extracts

The concentration of two main polyphenols, namely chlorogenic acid and luteolin-7-O-glucoside, in the micellar extracts was determined using HPLC chromatography with a UV-Vis detector (Knauer). The chromatographic separation was performed with Eurospher 100-3 C18 column (5 μm, 250 × 4 mm). Samples of 20 μL were eluted at 25 °C using isocratic flow of 1 mL/min of a solvent consisting of aqueous 0.05% formic acid (A) and pure acetonitrile (B) at a 2:8 volume ratio. The chromatographic separation was monitored with a UV-Vis detector at λ = 280 nm. The retention times of polyphenols were found based on pure standards for which standard curves in the range 0.01 to 1 mg/mL were plotted (R^2^ was 0.9946 and 0.9985 for chlorogenic acid and luteolin-7-O-glucoside, respectively). The results of triplicate analyses were expressed in mg/dm^3^ of extracts. Solvents of HPLC-grade and deionized water were used.

### 2.5. In Vitro Antioxidant Assay

The flavonoids content in the extracts were determined using their ability to react with aluminium chloride according to the previously used method [16]. The succeeding procedure was applied: 1 mL of the extract was diluted in 5 mL of distilled water and 0.3 mL of NaNO_2_ solution (5%) and left for 5 min. Next, 0.6 mL of AlCl_3_ solution (10%), and after 6 min, 2 mL of 1 M NaOH, were mixed. The obtained mixture was diluted with 1.1 mL of distilled water and the absorbance of the sample was measured directly, at λ = 510 nm. The UV/Vis spectra were recorded with Macherey Nagel Nanocolor spectrophotometer. The results were given as luteolin content (mg/dm^3^).

The total polyphenol concentration in the extracts was determined with the Folin-Ciocalteau (FC) colorimetric assay [16]. The procedure included the subsequent steps: 1 mL of the extract was mixed in 5 mL of the FC reagent for 4 min. Next, 4 mL of 7.5% sodium carbonate was added and so prepared solutions were stored without light for 2 h. After that, the absorbance of the samples was recorded, at a wavelength λ = 765 nm. The results were also given as rutin content (mg/dm^3^).

### 2.6. Particle Size Measurement and Zeta Potential Determination

The Dynamic Light Scattering (DLS) method was used to measure the hydrodynamic diameter and poly-dispersity of surfactant aggregates. The measurements were performed using the Litesizer™500 particle analyzer from Anton Paar (Graz, Austria) with the detection angles of 15°, 90°, and 175°, and equipped with a semiconductor laser (40 mW, 658 nm). Determination of the solutions’ ζ-potential was carried out at a scattering angle of 15°. Before measurements, all samples were filtered using a filter with a pore size of 0.45 μm, directly to the optical cell, to remove any impurities, or in case of extracts to refuse large aggregates. Measurements were performed at 293 K and at 1% concentration of surfactants, which was more than 100 times greater than the CMC. Each value was obtained as the average of three tests with, at least, sixty runs. The Anton Paar Kaliope was used to record and analyze data.

### 2.7. Computational Details

The self-aggregation of a series of non-ionic surfactants in aqueous solutions and solubilization of flavonoid into micelles was theoretically examined using a molecular dynamic method. Six models of surfactants, with systemically growing polyoxyethylene parts, were used: mono-, di-, tri-, tetra-, penta- and deca-ethylene glycol decyl ether. Cynaroside (luteolin-7-O-glucoside) was selected as a representative of flavonoids found in the *Bidens tripartite* extract [6,12]. The three-dimensional structures of surfactants and cynaroside were prepared in two steps: (i) conformational analysis by the InstantJChem [15] tool using Dreiding Force Field, (ii) the DFT optimization in Gaussian 16 [17] using the CAM-B3LYP [18] method in combination with the 6-31G(d, p) basis set [19,20,21,22,23] including water as a solvent in the PCM method [24].

Simulation boxes were prepared using Packmol [25,26] so the number of surfactants was equal to 50. The boxes were filled with water (TIP3P model) to obtain densities equal to 1000 g/dm^3^. Simulations of spontaneous aggregation of surfactants in aqueous solution were carried out in Gromacs 2016-s [27,28,29] using periodic boundary conditions in the GAFF force field, and consisted of four steps: minimization of energy, the equilibration: 100 ps isothermally, 100 ps isothermal-isobaric and finally appropriate simulation: 100 ns NPT with 2 fs steps. Simulations of the spontaneous solubilization of the flavonoid were performed according to the same scheme. The starting position of the flavonoid was random, with at least 10 angstroms from the micelle surface. Simulations were performed in triplicate, and the results were the arithmetic average while the structures were the result of clustering.

## 3. Results and Discussion

### 3.1. Extraction

In our previous work, the polyethoxylated/polypropoxylated fatty alcohols with HLB values in the range 6.3–8.6 were used to extract the *Bidens tripartite* [6]. In this study, a subset of more hydrophilic and commercially available surfactants from the same class was used (HLB in the range 9.7–16, Table 1, See Supplementary Materials Figure S1). The chosen compounds are applicable mainly in the cosmetics industry as o/w-type emulsifiers or solubilizers. However, the main goal of this study was to investigate the effect of their structure on the solubilization of flavonoids, which are efficient, attractive and eco cosmetic antioxidants. The surfactants’ physicochemical properties (Table 1) indicated that besides O5 they are highly hydrophilic surfactants with very good surface properties and low CMC values. Moreover, from this set, only O5 and possibly O10 could potentially be used in CPE technology due to the cloud point below 50 °C.

So far, the results revealed that non-ionic surfactants belonging to polyethers of fatty alcohols mainly affect the efficiency of flavonoid extraction from plant material [5,6,12,16]. The extremely effective eluent for flavonoids extraction from *Bidens tripartite* was the aqueous solution of C9-11 Pareth-5 (Rokanol^®^NL5, HLB = 8.6), i.e., the most hydrophilic among the studied Rokanols^®^. In the case of sourcing of polyphenols essences, any overall trend was not observed, but still, the content of the actives in NL5 concentrated extracts was higher than in other cases [9]. For calendula flowers, the solution of C9-11 Pareth-5 (C9/11E5) was also the most effective medium for polyphenols extraction. On the other hand, the highest concentration of flavonoids was received when more hydrophobic PPG-6 Steareth-7 (C18E7P6) was used. It was concluded that the polyphenol content decreases with the decreasing number of PEG/PPG groups in the surfactant structure. In the case of the antioxidant activity and the flavonoid content, there was no such relationship [5]. Since the dependence of the UAMME extraction yield on the surfactant structure has not yet been clearly explained, for further studies, we selected compounds with different carbon chain lengths but the same polyether group (two subsets: O20, S20, CS20 and CS12, T12, LT12 in Table 2) and with a diverse number of oxyethylene units but a fixed alkyl chain length (O5, O10, O20 in Table 2). Generally, aqueous surfactant solution utilization was unquestionably more efficient than water or ethanol for the extraction of polyphenols, therein flavonoids, from plant material. The highest total polyphenol content was determined in extracts with Oleth-10 and Oleth-5, while the highest total flavonoid concentration was obtained for Oleth-5. However, quantitative analysis revealed that the highest concentrations of both chlorogenic acid and luteolin glucoside were found in the extract with Ceteareth-12. These three surfactants are the least hydrophilic of the study (HLB equals to 9.7, 12.9 and 13.4 for O5, O10 and CS12, respectively).

Based on the results in Table 2, both structural modifications, i.e., oxyethylene unit number and length of the hydrocarbon chain affected the efficiency of the extraction process. Along with the decrease in the number of oxyethylene groups, the concentration of chlorogenic acid and luteolin glucoside increased, which was consistent with the total content of flavonoids. The unsaturation of the hydrocarbon chain slightly reduced the flavonoids extraction yield. It was associated with the very similar HLB value of considered O20 and S20. The results for the subset1 (surfactants contained 20 moles of oxyethylene) show that as the carbon chain lengthened, the content of chlorogenic acid and luteolin glucoside decreases. Again, this was consistent with the total flavonoid content. Furthermore, surfactants containing saturated alkyl chains were more effective eluents. Opposite findings for the second subset, where the number of OE groups was 12, were observed. Yields of both polyphenols and flavonoids were decreasing in a series of CS20 > T12 > LT12 extracts. Therefore, in this case, the increase of hydrocarbon chain length improved solubilization of active substances. The other authors had similar doubts. For example, Vinarov et al. [30] studied the solubilization of highly hydrophobic drugs in surfactant solutions. Studies illustrated that surfactant molecular structure could have a different effect on the solubilization capacity, but it depends mostly on the type of drug. They received the highest drug solubility by using surfactants with longer chain length. Similar studies were carried out for the phenol molecule as a water waste ingredient model [11]. Authors concluded that the phenol solubilization depends only on EO number, not on the carbon chain length of alcohol ethoxylates.

Summing up, the obtained results confirmed that micelle-mediated extracts from *Bidens tripartite* show a higher concentration of actives than water and ethanol extracts. It has been confirmed that the structure of the surfactant has an impact on the polyphenols’ extraction efficiency from plants. Current studies, together with previous ones, gave a more complete picture of this relationship. It can be concluded that the most optimal in the UAMME was ethoxylated fatty alcohol, where the ratio of the EO units to CH_2_ groups is in the range of 1.8–2.0. In our case, the criteria met two surfactants C9-C11 Pareth-5 and Oleth-10. Linking this to the hydrophobicity index gives us the HLB range from 8.6 to 12.9. However, this requires more studies on other structurally different surfactants.

### 3.2. Particle Size and ξ-Potential Determinations

The very useful tool for the monitoring of structural changes in colloidal solutions is the dynamic light scattering (DLS) measurement (Table 3). Solubilization of *Bidens tripartite* polyphenols into solutions of other surfactants was monitored by DLS in our previous work [6]. Increasing micelle size and neutralization of their surface because of polyphenols extraction into surfactants’ micelles was noticed. The most significant enlarging of micelle size was observed for the surfactant with the best solubilization capacity, C9-C11 Pareth-5. Similarly, in these studies, MME extraction was accompanied by an increase in aggregate size with simultaneous reduction of zeta potential and enhancement of the solution conductivity. Except for the 1% aqueous solution CS12, all eluents were initially nano-micellar systems. In the case of O5, a slightly larger aggregate size was caused by a low cloud point.

In addition, the temperature effect on the particle size in aqueous surfactant solutions was investigated (Table 4). Only O5 and CS20 showed stability over the entire temperature range. Interestingly, this coincided with extract measurements, where only these solutions remained nano-micellar.

### 3.3. Molecular Dynamic Simulations

So far, the results showed that the solubilization of polyphenols was a three-step process with relatively slow diffusion to the surface of the micelles, following the adsorption, and rather rapid inclusion into the micelle structure. The amphiphilic nature of polyphenols forced compounds to place them between the hydrophilic shell and the hydrophobic core of micelle. Additionally, the structure of the polyphenol molecule rather than its logP value determined their exact arrangement into the micelles [5,6]. To assess the effect of the number of oxyethylene groups in the surfactant structure on the flavonoid solubilization, five models of ethoxylated decyl alcohol derivatives (Table 5, Supplementary Materials Figure S2) were evaluated. They differed in hydrophobicity over a wide range, however, only C10E5 could mimic a commercially available Rokanol^®^ NL5 from the PCC Group. Based on the HLB values, calculated with the Griffin method, the first compound should be classified as w/o-type emulsifiers, the next two could be considered as o/w-type emulsifiers, while the last three could be categorized as solubilizers.

**Figure 1:**
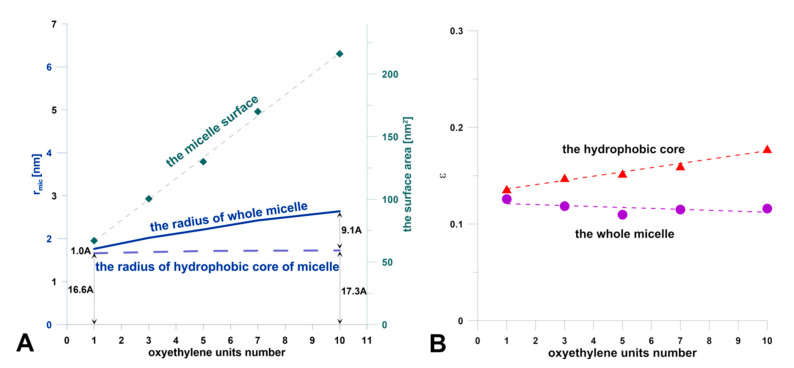
Geometrical features of micelles. (**A**) The micelle radius, the radius of the hydrophobic core of micelles, and the size of the surface area of the micelles (calculated as the solvent-accessible surface area) in the function of the oxyethylene group number. (**B**) The eccentricity (ε) of micelles in the function of the oxyethylene group number, defined as ε =1 − I_min_/I_avg_, where I_min_ is the moment of inertia along the principal axes with the smallest magnitude and I_avg_ is the average of all three moments of inertia.

Theoretical studies were carried out in two stages. At the beginning, simulations of molecule self-aggregation in aqueous solution were performed. The results indicated that all model compounds aggregate in water. Except for C10E15, which was too hydrophilic and was omitted in further analysis, they formed a single micelle made of 50 molecules. Obviously, the size of the surfactant aggregate increased with an increasing number of oxyethylene segments (Figure 1A). The micelle surface area grew even faster (Figure 1B). Based on the calculated moments of inertia of the aggregates, along the three main axes, their eccentricity was estimated. This parameter determines the sphericity of the micelles and assumes values close to zero for the spherical shape. In this study, all created micelles were spherical. Recent studies show that the eccentricity of micelles can be a theoretical indicator of colloidal solution stability [31]. Based on the calculated values (Figure 1B), it can be assumed that all surfactant solutions should be stable. Despite this, along with the increase in the number of EO groups, the centricity of the hydrophobic core of the micelles increases, which may be the first symptom of a decrease in the stability of such a colloidal system.

**Figure 2:**
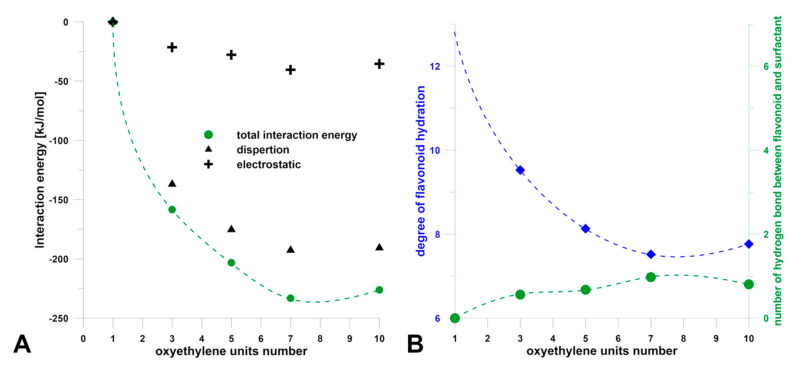
(**A**) The calculated average energy of interaction between luteolin-7-O-glucoside and surfactant in the function of the number of oxyethylene groups of surfactants; (**B**) The average degree of hydration of luteolin-7-O-glucoside and the average number of hydrogen bonds between flavonoid and surfactants in the function of the number of oxyethylene groups of surfactants.

In the second step, simulations of luteolin-7-O-glucoside (the chemical structure at Figure S2 of Supplementary Materials) solubilization in micellar solutions of model surfactants were carried out. Except for the most hydrophobic compound (C10E1), spontaneous adsorption of flavonoid on the micelle surface occurred during the simulation, which led to a single mixed micelle being formed. An example of the molecular structure of the flavonoid-micelle complex after 100 ns simulation is shown in Figure S3 of Supplementary Materials. To characterize the systems, the interaction energies between luteolin glucoside and surfactants were calculated. Furthermore, the number of hydrogen bonds formed in these complexes, as well as the decrease in the hydration degree of luteolin glucoside, were studied (Figure 2). The results indicated that the stabilization energy of the micelle-flavonoid complex increased markedly with the increase of surfactant hydrophilicity until it reached the minimum at the seven oxyethylene groups in the surfactant. The most hydrophilic (C10E10, HLB = 15.28) again formed a slightly less stable complex. Both components of energy, i.e., dispersion and electrostatic contributions, were evolved in the same way. However, the main contribution to the total energy has a dispersion term. These results confirmed that the main driving force for flavonoid solubilization is hydrophobic interactions. This was also revealed by the small number of hydrogen bonds formed between the glucoside and micelle. Moreover, the incorporation of luteolin-7-O-glucoside into the surfactant micelle was accompanied by a significant decrease in its degree of hydration, which was the greater hydrophilic surfactant. As with the energy plot, a minimum was reached when seven oxyethylene units were achieved.

Based on the theoretical considerations above, it can be presumed that the best extraction medium should be the hypothetical surfactant C_10_H_21_(OC_2_H_4_)_7_OH. These observations are consistent with the results of experimental studies, where medium hydrophilic surfactants like Rokanol^®^NL5 or Brij^®^O10 were proven to be better eluents. This fully supports structural recommendations from the experimental part. Some other authors proposed, for practical cloud-point extraction of phenol from the wastewater, alcohol ethoxylate surfactants with low EO number (e.g., 5) and a normal commercial range of alkyl carbon numbers (9–13) [11]. Therefore, we also showed here the energetic and molecular proof for these findings from 10 years ago.

## 4. Conclusions

In this work, a connection between the chemical structure of non-ionic surfactants and the efficiency of the extraction process was found. The effect of hydrophobic chain length and number of oxyethylene groups on the quality of three-part beggarticks (*Bidens tripartita*) extracts was investigated. It turned out that the efficiency of solubilization depends simultaneously on both structural modifications of surfactants. Based on experimental and theoretical studies, we proved that the surfactant must have a certain optimal ratio of hydrocarbon chain length to the number of OE groups. For the UAMME of plant material, we recommend the use of ethoxylated fatty alcohols, where the above index is in the range of 1.8–2.0.

Due to its excellent efficiency, environmental friendliness, low costs, and waste-free process, the ultrasonic assistance micelle-mediated extraction method seems to be one of the best available technologies to obtain flavonoid-rich extracts.

## Figures and Tables

**Table 1:** Characteristics of the surfactants.

Surfactant INCI	Acronym	Type of Alcohol	OE	Cloud Point [°C]	Surface Tension [mN/m]	HLB ^2^	CMC [mg/dm^3^]
Oleth-5	O5	C_18:1_	5	16–22 ^1^	30	9.7	9.3
Oleth-10	O10	C_18:1_	10	47–55 ^1^	31	12.9	19.0 ^1^
Oleth-20	O20	C_18:1_	20	>100 ^1^	32	15.6	25.0 ^1^
Steareth-20	S20	C_18_	20	73–77 ^1^	45	15.6	25.0 ^1^
Ceteareth-20	CS20	C_16_/C_18_	20	>100 ^1^	37	16.0	55.6
Ceteareth-12	CS12	C_16_/C_18_	12	89	35	13.4	18.3
C12-C15 Pareth-12	T12	C_12_/C_15_	12	96	31	14.4	22.3
C12-C13 Pareth-12	LT12	C_12_/C_13_	12	86	29	15.0	35.0

^1^ provided by the manufacturer, ^2^ calculated with the Griffin method using InstantJChem.

**Table 2:** Characteristics of UAMME, water and ethanol extracts.

Extraction Medium	Number of CH_2_ and OE Units	Total Polyphenols Content per Rutin [mg/dm^3^]	Total Flavonoids Content per Luteolin [mg/dm^3^]	Chlorogenic Acid [mg/dm^3^]	Luteolin 7-O-Glucoside [mg/dm^3^]
O5	18:1; 5	424.9 ± 1.1	83.2 ± 2.1	43.9	23.1
O10	18:1; 10	469.3 ± 10.0	72.2 ± 0.3	25.3	11.3
O20	18:1; 20	339.2 ± 0.8	53.1 ± 0.7	25.4	10.2
S20	18; 20	334.1 ± 0.8	62.2 ± 8.5	39.8	18.2
CS20	16/18; 20	334.1 ± 0.5	56.3 ± 1.6	40.3	20.2
CS12	16/18; 12	253.5 ± 2.5	75.5 ± 1.2	66.4	28.5
T12	12/15; 12	301.5 ± 15.5	68.4 ± 0.3	59.6	20.3
LT12	12/13; 12	371.6 ± 8.9	62.9 ± 0.5	55.0	13.8
Water	-	34.0 ^1^	25.6 ^1^	9.0	0.44
Ethanol	-	40	16	0.2	3.5

^1^ Ref. [6].

**Table 3:** Micelle’s size and Zeta potentials of 1% surfactant solutions and extracts.

Surfactant	Hydrodynamic Diameter ^1^ [nm]	PDI [%]	Size Distribution by Intensity [nm] ^2^			ξ [mV]	Conductivity [mS/cm]
			1st Peak/%Intensity	2nd Peak/%Intensity	3rd Peak/%Intensity		
O5	110.3 ± 5.0	25.9 ± 1.6	141.6/100	-	-	−9.4 ± 0.4	0.049
O10	10.9 ± 0.4	20.2 ± 1.9	9.5/84	429.0/16	-	−5.3 ± 1.8	0.061
O20	11.9 ± 0.3	23.3 ± 1.4	9.1/70	4517/24	270/6	−19.2 ± 1.5	0.066
S20	12.4 ± 0.7	22.9 ± 1.9	8.6/55	6471/35	623/10	−14.9 ± 5.2	0.031
CS20	9.5 ± 0.3	8.5 ± 5.1	9.7/100	-	-	−19.2 ± 5.4	0.030
CS12	852.9 ± 33.2	22.8 ± 2.9	890.4/97	41.6/3	-	−5.2 ± 0.3	0.068
T12	32.4 ± 38.7	23.2 ± 1.7	7.4/64	208.0/16	3206/20	−13.8 ± 1.2	0.051
LT12	218.7 ± 124	37.8 ± 14.0	7.0/40	440.2/50	3859/10	−19.0 ± 4.3	0.037
extracts							
O5	79.2 ± 2.3	24.9 ± 1.1	97.0/100	-	-	−1.6 ± 0.1	1.961
O10	358.2 ± 44.1	24.3 ± 1.2	447.7/60	16.4/40	-	−2.5 ± 0.2	2.468
O20	414.4 ± 70.7	32.1 ± 2.5	329.5/61	5713/22	9.2/17	−3.3 ± 0.1	2.213
S20	4658 ± 3659	35.3 ± 9.5	370.7/50	6966/35	9.7/15	−3.3 ± 0.0	1.993
CS20	18.4 ± 0.2	21.0 ± 1.9	340.7/54	9.8/46	-	−2.8 ± 0.5	1.556
CS12	4838 ± 1205	28.5 ± 2.2	3067/94	1612/4	55.3/2	−2.8 ± 0.1	2.108
T12	1188 ± 683.1	27.3 ± 9.1	525.7/60	4910/34	8.4/6	−5.7 ± 0.4	2.034
LT12	1102 ± 87.8	58.5 ± 2.3	2508/63	391.0/30	11.2/7	−4.4 ± 0.1	2.159

^1^ measured by the back-scattering mode, ^2^ intensity = relative frequency intensity weighted.

**Table 4:** The size of micelles under different temperatures.

Surfactant	Hydrodynamic Diameter [nm]/Temperature [°C]				
	10	25	30	40	50
O5	107.3	112.3	111.0	110.7	111.3
O10	9.4	12.3	16.9	28.3	47.9
O20	12.6	102.3	359.5	953.4	13.4
S20	12.2	237.1	179.0	399.4	590.7
CS20	9.9	11.1	10.7	10.7	10.5
CS12	927.2	1458	1337	858.5	12.2
T12	155.2	55.7	118.6	129.5	104.9
LT12	9.1	10.9	10.3	142.0	148.1

**Table 5:** The studied surfactant models and calculated HLB indices.

Surfactant Model	Denotation	HLB_ChemAxon_	HLB_Griffin_
C_10_H_21_OC_2_H_4_OH	C10E1	5.97	6.04
C_10_H_21_(OC_2_H_4_)_3_OH	C10E3	9.22	10.27
C_10_H_21_(OC_2_H_4_)_5_OH	C10E5	11.69	12.54
C_10_H_21_(OC_2_H_4_)_7_OH	C10E7	13.81	13.95
C_10_H_21_(OC_2_H_4_)_10_OH	C10E10	16.69	15.28
C_10_H_21_(OC_2_H_4_)_15_OH	C10E15	21.10	16.55

## Supplementary Materials

The following are available online at https://www.mdpi.com/2079-4983/11/3/57/s1, Figure S1: Chemical structures of surfactants used in the experimental part; Figure S2: Chemical structures of model surfactants (polyoxyethylene ethers of decyl alcohol) and luteolin-7-O-glucoside used in the MD simulations; Figure S3: The interaction pattern of luteolin-7-O-glucoside with surfactants (C10E7) and waters in simulated micellar solution.
