# Supplementary material for: The Accumulated Effect of the Number of Ethylene Oxide Units and/or Carbon Chain Length in Surfactants Structure on the Nano-Micellar Extraction of Flavonoids
Source: J Funct Biomater. 2020 Aug 7;11(3):57. doi: 10.3390/jfb11030057 (PMC7563964; doi:10.3390/jfb11030057)
Supplement: Supplementary file 1 [file jfb-11-00057-s001.pdf]

Journal of *Functional Biomaterials*

Supplementary Materials

The Accumulated Effect of the Number of Ethylene Oxide Units and/or Carbon Chain Length in Surfactants Structure on the Nano-Micellar Extraction of Flavonoids

Karolina Śliwa and Paweł Śliwa *

Faculty of Chemical Engineering and Technology, Cracow University of Technology, 24 Warszawska St., 31-155 Cracow, Poland; karolina.sliwa@pk.edu.pl

* Correspondence: pawel.sliwa@pk.edu.pl; Tel.: +48-12-628-27-58

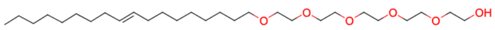

Oleth-5

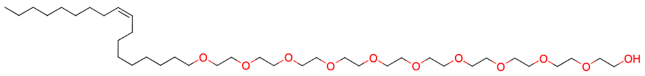

Oleth-10

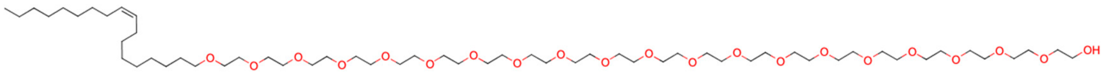

Oleth-20

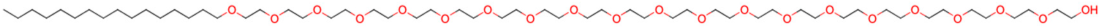

Palmiteth-20

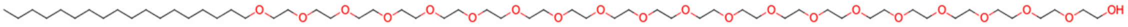

Steareth-20

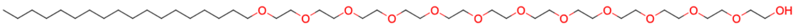

Steareth-12

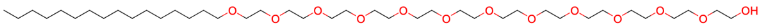

Palmiteth-12

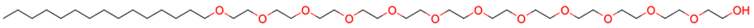

C15 Pareth-12

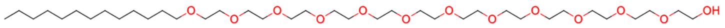

C13 Pareth-12

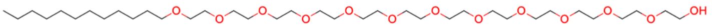

Laureth-12

Figure S1. Chemical structures of surfactants used in the experimental part. Ceterath-20 is equimolar mixture of Palmiteth-20 and Steareth-20, Ceteareth-12 is equimolar mixture of Palmiteth-12 and Steareth-12, C12-C15 Pareth-12 is mixture of Laureth-12, C13 Pareth-12 and C15 Pareth-12, and C12-C13 Pareth-12 is mixture of Laureth-12 and C13 Pareth-12.

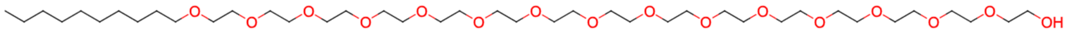

C10E15

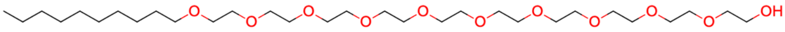

C10E10

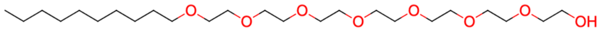

C10E7

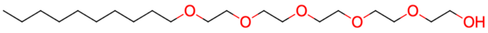

C10E5

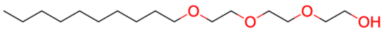

C10E3

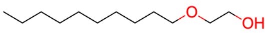

C10E1

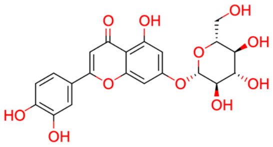

Luteolin-7-O-glucoside

Figure S2. Chemical structures of model surfactants (polyoxyethylene ethers of decyl alcohol) and luteolin-7-O-glucoside used in the MD simulations.

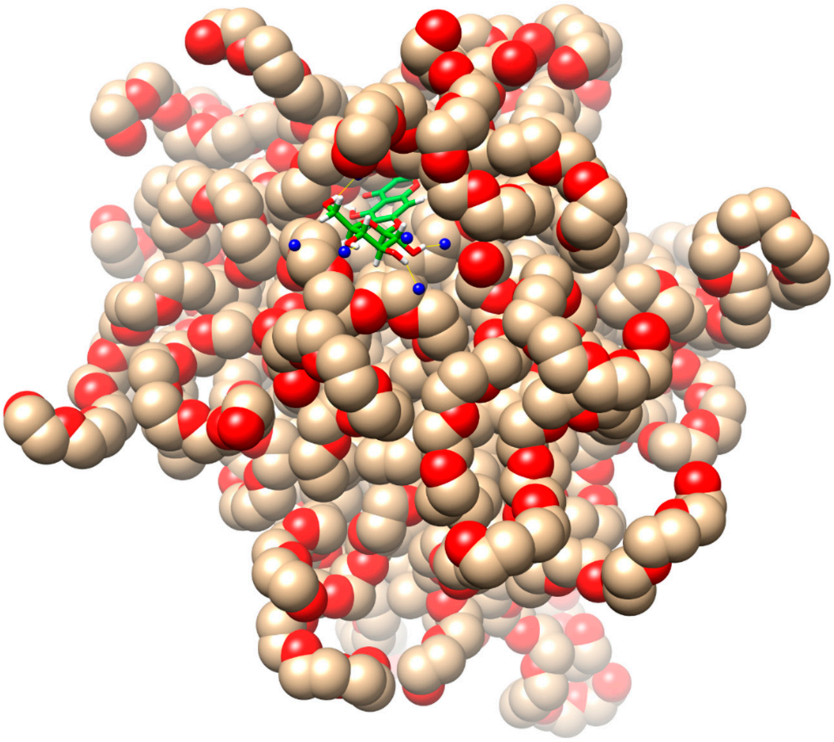

Figure S3. The interaction pattern of luteolin-7-O-glucoside with surfactants (C10E7) and waters in simulated micellar solution. Surfactants are marked as spheres with sand-coloured carbons and red oxygens, but hide all hydrogens. Flavonoid is marked with green sticks. Hydration waters of flavonoid are blue spheres located in the positions of oxygen atoms. Hydrogen bonds are marked with yellow dash lines.
